# Using secondary prevention strategies in patients after PCI: a narrative review

**DOI:** 10.3389/fpubh.2025.1562201

**Published:** 2025-07-10

**Authors:** Guangdi Xi, Fuguo Yang, Jianan Xu, Jingzhe Liu, Dexin Chen

**Affiliations:** Qingdao University School of Nursing, Qingdao University, Qingdao, China

**Keywords:** percutaneous coronary intervention, secondary prevention, postoperative rehabilitation, self-management, health behavior change

## Abstract

**Objective:**

The objective of this review is to integrate the content and effectiveness of secondary prevention interventions in post-PCI patients so that provide direction for the selection of more effective secondary prevention measures in the future.

**Method:**

A narrative review was performed, which included a literature search without language and study design restrictions in PubMed, Embase, and MEDLINE from January 1, 2000, to May 15, 2023. Search terms included free-text words for the key concepts of “Percutaneous Coronary Intervention” “PCI” and “secondary prevention”.

**Results:**

This study consolidated the measures of secondary prevention included in the studies and found that patient education strategies had the highest rate of use (78%), followed by exercise (56%), with smoking cessation, diet, blood pressure control, and medication also being used in the remainder. We critically analyzed these secondary prevention strategies and found that only 56% of these secondary preventions were effective in their use, with the remaining 44% having non-significant differences between the intervention and control groups. 44% of the studies incorporated mHealth, and mHealth had a facilitating effect on the intervention. We found that patients had better results on subjective measures after surgery (*p* < 0.05), while none of the objective measures were significant.

**Conclusion:**

This study found that the use of secondary prevention in post-PCI patients is not limited to medication; five non-pharmacologic measures, namely patient education, exercise, dietary modification, blood pressure control, and smoking cessation, have also been actively used. Some studies have combined mHealth technology with secondary prevention measures with good results, in which the interactivity of mHealth should be focus on. However, changes in objective prognostic measures after the application of secondary prevention measures in post-PCI patients were not significant, indicating that the efficacy of the measures was not significant, which is an issue that deserves to be emphasized in subsequent studies. Meanwhile, educational, economic, and social support challenges in the older adult population may hinder the effective implementation of secondary prevention, future studies in the older adult population should prioritize addressing these issues to optimize the prognosis of older adult patients.

## 1 Introduction

According to established guidelines (1), coronary heart disease is associated with significant morbidity and mortality on a global scale. Factors such as the accelerated aging of the population and the widespread prevalence of unhealthy lifestyles have contributed to coronary heart disease consistently having the highest mortality rate among cardiovascular diseases in both urban and rural areas (2). In the aftermath of the COVID-19 pandemic, issues related to postoperative rehabilitation and complications in patients with coronary artery disease have become increasingly prominent, imposing a substantial disease burden on society (3).

Percutaneous Coronary Intervention (PCI) is a well-established and efficacious invasive procedure designed to compress atherosclerotic plaques and expand the luminal diameter of stenotic coronary arteries. This is achieved by introducing a catheter to the site of narrowing and inflating its distal end (4). PCI offers the benefits of minimal trauma and rapid recovery (5). As a strategy intended to slow disease progression and enhance patient recuperation, PCI has become one of the most commonly utilized therapeutic interventions for coronary artery disease (5, 6). The global incidence of patients undergoing percutaneous coronary intervention (PCI) is projected to exceed 1.1 million (7). Individuals undergoing PCI are at risk for PCI-related myocardial injury. Consequently, it is imperative to continue addressing coronary heart disease-associated risk factors, including hypertension, obesity, diabetes mellitus, elevated blood cholesterol, and cigarette smoking. This necessitates collaborative efforts between healthcare professionals and patients to enhance patients’ self-management capabilities through systematic interventions aimed at improving prognosis and preventing recurrence (8, 9). Secondary prevention of coronary heart disease (10) are intended to promote a positive mental state and a healthy lifestyle, thereby reducing the risk of recurrent cardiovascular events and sudden cardiac death. Furthermore, secondary prevention strategies (11–13) emphasize the stringent control of risk factors in patients with pre-existing coronary heart disease to avert the recurrence of cardiovascular events and the onset of heart failure. This approach represents an optimized strategy for the prevention and management of coronary heart disease at the current stage.

However, what secondary prevention strategies have been focused on and used in post-PCI patients during cardiac rehabilitation, how effective are their interventions, and what role does it play in public health? In our current understanding, no study has addressed this issue for patients after PCI. Therefore, the study aims to review the content of secondary prevention interventions and intervention effects in post-PCI patients and to analyze the factors influencing them. The syntheses obtained from a targeted and critical-thinking approach could provide the basis for future systematic syntheses and provide direction for the selection of more effective secondary prevention measures in the future.

## 2 Methods

A narrative review was performed. This type of review provides a flexible approach in the analysis and interpretation of the literature (39). To guide our search strategy, we employed the PICOs criteria encompassing participant, intervention, comparison, outcome, and study (14). Additionally, the Preferred Reporting Items for Systematic Reviews and Meta-Analysis (PRISMA-ScR) (15) was used to structure both the research reporting and the presentation of results.

We conducted a comprehensive search for relevant studies from the inception of the database to May 1, 2025 were searched, using the following databases: PubMed, WOS (MEDLINE), Embase, Cochrane, and CINAHL. The search strategy for this study was carefully designed according to the PICOs framework, and search queries were tailored to each database consulted. The keywords used were “Percutaneous Coronary Intervention OR PCI” AND “secondary prevention OR early therapy OR relapse preventions” AND “randomized controlled trial OR random allocation.” Reference lists from relevant articles were hand-searched for additional relevant papers.

To be included, studies had to (1) focus on adults (aged >18 years) (2)The study conducted at least one postoperative secondary prevention strategy for patients with coronary PCI and (3) the articles were in English but there were no yearly restrictions. We have also focused our analysis on studies carried out in randomized controlled trials so that evaluated the effects of secondary prevention interventions after PCI from published studies. Exclusion criteria included studies unrelated to public health, studies published in languages other than English, and studies lacking full-text availability. The researchers used a two-person (G. D. X., J. N. X.) back-to-back search to ensure the inclusion of diverse and comprehensive evidence wherever possible. A third reviewer (F. G. Y.) was involved if consensus could not be reached.

## 3 Result

### 3.1 Study selection

**Figure 1:**
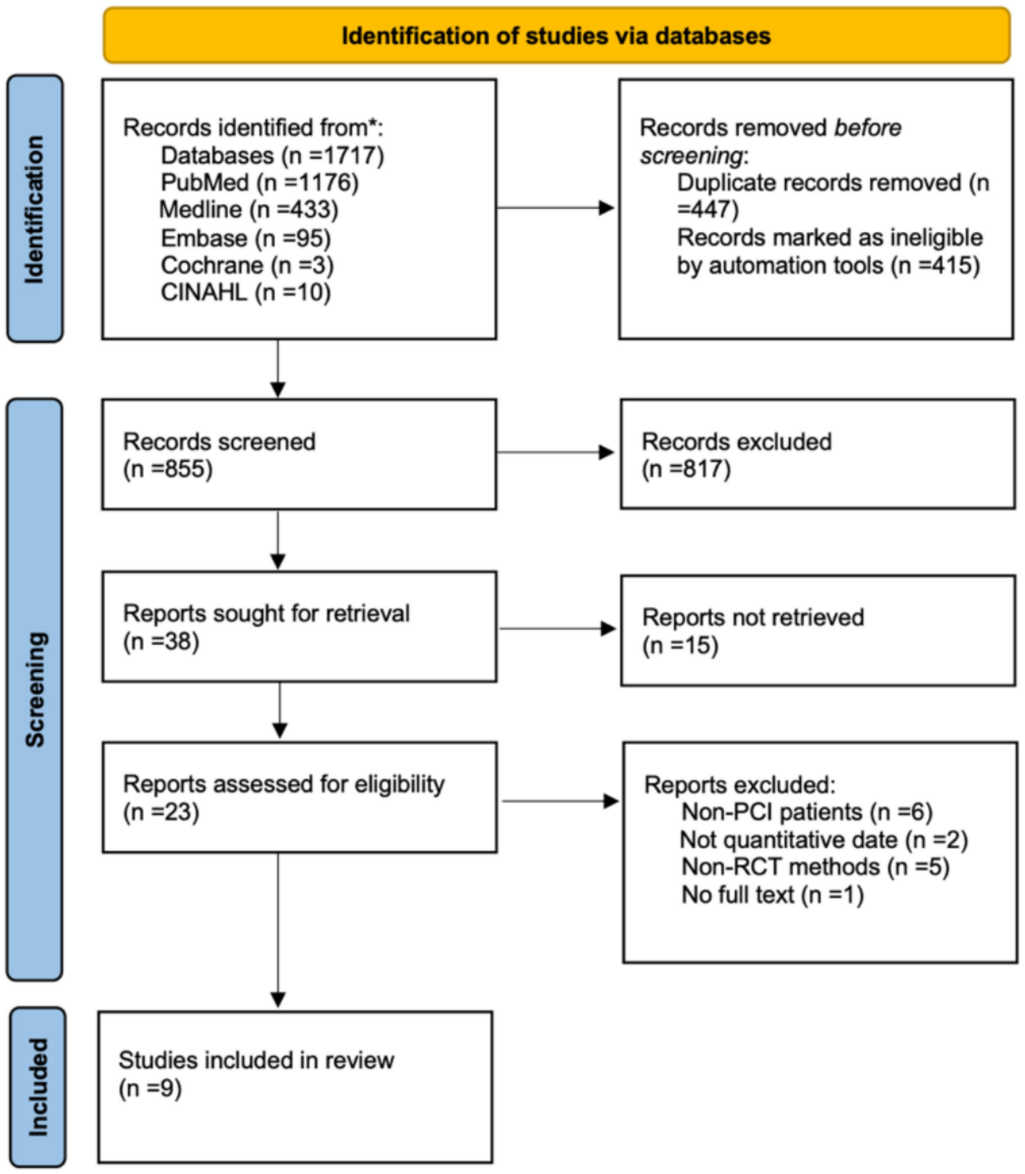
The flowchart of literature search.

As described in Figure 1, the literary search initially identified 1717 papers from five base stores. A total of 9 studies (16–24) were selected for data extraction. The total number of people included in the study was 2,252 (mean population size, 250.2 patients; median, 225 patients), most studies did not distinguish between men and women. The majority of patients were 18 years of age or older.

### 3.2 Study characteristics

The studies included in this review span a period of 18 years, with the earliest publication dating back to 2005 and the most recent to 2023. Most studies were published in the 2020–2023 (16–18, 21, 23) timeframe, accounting for 50% of the total (5 studies). This was closely followed by articles published in 2010–2014 (19, 20, 24, 25), which accounted for 40 per cent of the total (4 studies), with the remaining article published in 2005 (22).

The studies were conducted across various regions of the world, with the Nordic region (16, 18, 22, 23) having the highest number of published studies, accounting for 40% of the total (4 studies), The Americas (20, 21, 24) contributed 30% (3 studies), while Australia (19, 25) and Asia (17) had fewer studies, together accounting for 20% of the total (Each contributed one study).

The articles we chose for this study were from academic journals and they all were quantitative studies, using randomized controlled methods for the trials. To ensure the smooth running of the trials, most of the studies were conducted using the principle of the randomized controlled semi-blind method.

The use of secondary prevention measures in this study (18–22) was mostly not limited to one, and the researchers used a combination of several interventions. The highest percentage of the number of uses was the use of one secondary intervention (60 per cent of the total, six articles), the next highest was the use of four secondary interventions (20 per cent of the total, two articles), and the same number of studies used two, three and five (all 10 per cent of the total, one article each). The studies that used multiple secondary interventions also mostly intervened and moderated other aspects based on the health promotion approach. Four studies integrated mHealth into secondary prevention measures for coronary heart disease (16–18, 20). The structure of the included studies is shown in Table 1, and the detailed content of the studies is shown in Table 2.

**Table 1:** Secondary prevention strategies used in the literature.

Included literature	Secondary prevention strategies used in the literature						m-health
	Education	Excise	Cigarette quitting	Diet	blood pressure control	Use of medications	
van Bakel et al. (18)							
Pittaet et al. (21)							
Bae et al. (17)							
Bruggmann et al. (16)							
Hägglund et al. (23)							
Turner et al. (19)							
Rinfret et al. (20)							
Devon et al. (24)							
Lisspers et al. (22)							

**Table 2:** Papers included in systematic literature review.

Country	Patient population (mela/%)	The type of secondary prevention used	m-health	Intervention	Primary outcome	Effectiveness of interventions
The Netherlands	237 (164/77%)	Excise	PPT presentations	SIT LESS program (18)	The change in device-based ST from pre-CR to post CR.	The SIT LESS intervention did not induce significantly greater reductions in sedentary time compared to controls.
Brazil	125	Education/Excise	/	An educational cardiac rehabilitation program, based on SCT (21)	Baecke-Habitual Physical Activity Questionnaire (BHPAQ)	—
Korea	879 (732/83.3%)	Cigarette quitting /Diet/Education/Excise	SMS	a 1-way SMS text messaging program (17)	The LDL-C level, SBP, and BMI	Physiological measures of the primary outcomes, including LDL-C levels, SBP, and BMI, were not significant.
Switzerland	60 (51/85%)	Education	Web-hosted video	“Mon Coeur, Mon BASIC” Web-Hosted Video (16)	ARMS Score	In the intervention group, significant increases in knowledge from baseline to 1 and 3 months, but not to 6 months.
Sweden	225	Blood pressure control	/	Ambulatory blood pressure measurement(ABPM) (23)	Ambulatory blood pressure (ABP)	ABPM did not lower blood pressure in patients with CAD apart from in those with elevated ABP.
Australia	275	Use of medications /Cigarette quitting /Diet/Education/Excise	/	Cognitive behavior therapy(CBT)and motivational interviewing (19)	Psychological functioning	—
Canada	300 (219/73%)	Education	Telephone interviews	Phone calls (20)	The number of days with available aspirin and clopidogrel (from pharmacy records)	—
America	64 (32/50%)	Education	/	Viewed the Know and Go! slide presentation (24)	a 20-item instrument Score	—
Sweden	87	Blood pressure control /Diet/Education/Excise	/	4-week residential stay at the intervention unit and 11-month structured maintenance phase (22)	Changes in OLCx areas	—

“/“indicates not applicable. “—“indicates “The results of the intervention were positive and aligned with the anticipated expectations.

### 3.3 Secondary prevention

In addition to appropriate drug therapy, a multidisciplinary behavioral approach should be taken to help patients achieve a healthy lifestyle, promote cardiac rehabilitation, and improve the quality of life after surgery. The most recent update of the ESC/AHA guidelines puts a strong emphasis on cardiovascular risk, lifestyle changes, and exercise interventions in patients with ACS (26). In addition to this, secondary prevention of coronary artery disease can be carried out in patients from Patient In addition to this, secondary prevention for patients with coronary heart disease can also start from Patient education, Key lifestyle interventions for risk-factor control (e.g., smoking cessation, weight loss, attention to diet, exercise, etc.). At the same time, the incremental value of telemedicine in secondary prevention should be emphasized, and the use of mHealth big data to improve patients’ adherence to medications and healthy lifestyles is crucial for intervention outcomes (26).

To clarify the current scope of use and intervention effectiveness gaps in secondary prevention after PCI, we consolidated the measures of secondary prevention included in the literature (see Table 1) and found that patient education strategies had the highest rate of use (78%), followed by exercise (56%), with smoking cessation, diet, blood pressure control, and medication also being used in the remainder. We critically analyzed these secondary prevention strategies and found that only 56% of these secondary preventions were effective in their use, with the remaining 44% having non-significant differences between the intervention and control groups, and we present the findings of the non-significant differences in Table 2. We conducted a stratified analysis of effective and less effective studies.

#### 3.3.1 Results on objective indicators

We found that the use of secondary prophylaxis had a poor effect on changes in objective measures of prognosis in patients after PCI, the result reported in the studies by Bae (17), van Bakel (18), and Hägglund (23). In their study, Bae et al. (17) found that most of the patients would control their risk factors according to the healthcare requirements, and there was a change in subjective behavior, but at the end of the intervention, the improvement in physiological indicators (including LDL-C levels, SBP, and BMI) was not significant. van Bakel et al. (18) found the same results, with an improvement in sedentary behavior in the control group after the “SIT LESS” strategy, but there was no statistically significant improvement in the outcome measures, and the team found no change in quality of life or self-management ability at the later 12-week follow-up, while Bruggmann et al. (23) also found that that there was no difference in patient medication adherence at 1 and 6 months postoperatively. Guidelines (27) recommend ABPM as an alternative hypertension diagnosis to OBP measurement, but Hägglund et al. (23) found no efficacy of ABPM in blood pressure control in patients with CAD, except in patients with ABP, to reduce blood pressure in patients with CAD, other than affecting changes in antihypertensive therapy.

#### 3.3.2 Results of subjective indicators

##### 3.3.2.1 Education

Education (16, 17, 19–22, 24)is currently the most used measure by researchers in trials, and in recent years it has begun to be combined with mHealth (16, 17, 20, 24) Pitta et al. (21) intervened in physical activity through educational programs and promoted behavior change using the “motivational” strategy in the behavior change technique at follow-up visits. The final mean BHPAQ total score was higher in the education program group (7.941.84) than in the conventional group (6.901.09), with a mean difference of 1.04 (95% CI: 0.34, 1.69). Bae et al. (17) and Bruggmann et al. (16) borrowed mobile medical methods to carry out education, using online platforms and mobile phones to carry out propaganda and education, and improve the efficiency of intervention, although the results were not statistically significant, but they were effective in improving self-management, and said that they would continue to promote the mobile medical education model in the future. Rinfret et al. (20) also applied the intervention model of mHealth combined with health education, but the outcome measure was mainly medication adherence. The large population base worldwide that lacks medication adherence has led to treatment failure (28), Rinfret et al. (20) used simple animated films freely available online as educational content, integrated to form the web-hosted video “Mon Coeur, Mon BASIC,” which provided a more accessible approach for patients. Outcome metrics were statistically measured on a self-assessment scale with good internal consistency, and although there was no difference in the scores of the intervention group and the control groups did not differ (13.52,95% CI 12.63–14.41 and 13.68,95% CI 12.96–14.76, respectively; *p* = 0.33), the median ARMS score was significantly more stable in the intervention group (~12) and health education was relevant for prolonging medication duration. Turner et al. (19) and DeVon et al. (24) integrated educational interventions with other secondary prevention measures to develop a multi-perspective intervention plan to conduct experiments in terms of diet, smoking cessation and improved lifestyle behaviors, while Devon et al. also restricted smoking, according to post-discharge follow-up data, the intervention group scored significantly higher than the standard treatment group (*F* = 15.21; *p* < 0.001) and the obvious intervention group had a large change in lifestyle, and all coronary events were significantly reduced. Devon et al. (24) emphasize the need for large-scale and long-term evaluations of this lifestyle-oriented secondary prevention intervention in the future.

##### 3.3.2.2 Exercise

Physical activity reduces the risk of many adverse health outcomes and risk factors in all ages and both sexes. There is an inverse relationship between moderate-to-vigorous physical activity and all-cause mortality, cardiovascular mortality, and atherosclerotic cardiovascular disease (ASCVD) (26). Although the results in the Turner et al. (19) cardiac program showed no between-group differences in activity change between the two groups, total activity hours increased significantly over the trial time (19). Pitta et al. (21) found that at the end of follow-up, the educational program group had a higher mean total BHPAQ score (7.94 ± 1.84) indicating performing more physical activity than the usual care group (6.90 ± 1.09), with a mean difference of 1.04 (95% CI: 0.34, 1.69). van Bakel et al. (18) conducted a 12-week nurse-guided mixed behavioral change intervention in addition to a comprehensive central cardiac rehabilitation program for 108 patients in the intervention group, combined with a pocket-worn activity tracker connected to a smartphone app for continuous monitoring of sedentary time. Although the between-group difference in sedentary time reduction did not reach statistical significance [−0.4 (−1.0 to 0.3) hours/day] the proportion of patients with post-rehabilitation sedentary time exceeding the upper limit of normal (≥ 9.5 h/day) was significantly lower in the SIT LESS program group than in the control group (48% vs. 72%), and the likelihood of being sedentary ≥ 9.5 h/day was reduced.

### 3.4 M-health

In the process of secondary prevention intervention, mobile medicine is also gradually used, and variable control is carried out through various methods such as SMS (17), telephone interviews (20), PPT presentations (18), video viewing (17) and professional mobile device monitoring (18). In van Bakel et al. (18), after the patients were taught a systematic PPT-related rehabilitation knowledge, the researchers strapped a pocket activity tracker to them and connected it to a smartphone app for monitoring, and if they were sedentary (≧ 30 min), the tracker would provide vibrating haptic feedback to remind the patient to get up and move. This intuitive and effective nudging mobile device reduced sedentary time in both groups, Before the cardiac rehabilitation program, the daily standing time(ST)was 11.3 ± 1.6 h/day in the control group and 10.9 ± 1.6 h/day in the SIT LESS group. After the cardiac rehabilitation program, the change in ST was −1.2 [95% confidence interval (CI) − 1.7; − 0.8] hours/day, control group and − 1.6 (95% CI − 2.1; − 1.1) hours/day.

Rinfret et al. (20) and Bae et al. (17) chose telephone follow-up and mobile phone text messaging as mobile interventions that can availably control costs. In addition, online video has also been chosen as a missionary path. Bruggmann et al. (16) chose a web hosting platform that is compatible with both mobile phones and tablets as a means of intervention, with the content of a simple animation that is freely available online. The video provides information on cardiac function and physiopathology of ACS, acute care for ACS (colonography and PCI), and medications (usefulness and side effects) prescribed after ACS, with a total length of about 15 min, which is more acceptable to older patients. The results showed that web-hosted video appeared to significantly improve medication adherence in patients with ACS within a few months of treatment initiation.

## 4 Discussion

### 4.1 The impact of secondary prevention measures on changes in objective indicators deserves sustained attention

The guidelines (26) emphasize the use of multidisciplinary behavioral approaches recommended to help patients time a healthy lifestyle and improve their prognosis, in addition to appropriate medication management. Risk perception is part of many of the major health behaviors that can be changed, so the adoption of a variety of preventive strategies such as education, exercise, etc. is then aimed at reducing the risk factors for recurrence associated with an individual’s cardiovascular risk, and the best measurements, in addition to those measured by scale tools, are objective clinical measures. However, Bae (17), van Bakel (18), and Hägglund (23) found that the use of secondary prevention measures had little effect on the changes in objective indicators of patient prognosis after PCI, including the most basic blood pressure and lipid profiles, which is something to think about. We think that this may be related to self-neglect in patients’ perception of the disease over time, van Bakel et al. found that quality of life and self-management of patients in the intervention group did not increase over time, and the authors questioned the sustained effectiveness of traditional CR measures in reducing sedentariness in patients, which is in line with a previous meta-study conducted by Dibben et al. (29), Bruggmann et al. also found that the patient intervention group did not produce a change in medication adherence even after 3 months, it also implies that the patient developed self-neglect in postoperative self-management after a certain period of time. The inability of self-neglectors to perform basic self-care tasks that threaten a person’s health and safety was confirmed in XinQi Dong’s study (30), but whether improving this leads to improvements in clinical objective measures in post-PCI patients’ needs further validation.

### 4.2 M-health is gradually used in conjunction with secondary prevention

In the results of this study, it was found that mHealth was gradually combined with the secondary prevention of coronary heart disease. This is a big step forward in the management of postoperative rehabilitation. Studies have shown that the use of mobile apps in healthcare has been identified as a powerful tool to promote behavioral change in patients with PCI, and recent studies (31) have found mHealth to be effective in changing patient health and preventing recurrence in different coronary artery disease populations. Another meta-analysis found that different modalities of mHealth were effective in changing patients’ lifestyle habits (32). M-Health was defined as the use of mobile technology to provide healthcare or health promotion, is one of the most promising new advances in healthcare technology (33). Studies have shown that the m-health is becoming an incentive for nurses to provide quality health care. The m-health can improve access to health care and meets patients’ expectations for health literacy (34, 35) so it is beneficial for patients to improve self-efficacy and prognosis. Among them, the interactive features of mHealth should be focus on.

### 4.3 Public health challenges of secondary prevention stages

Although the benefits of adopting healthy habits are well known, the vast majority of people with PCI do not meet the criteria for secondary prevention goals set out in international clinical guidelines (36). There are still a range of lifestyle, diet and exercise, and cardiovascular risk factor issues that need to be further optimized (37). The European Society of Cardiology (38) places therapeutic drug adherence and healthy lifestyle habits at the center of secondary prevention strategies for coronary syndromes, which is consistent with the conclusions reached in this review. There are not many studies of behavioral management using secondary prevention strategies in patients with PCI, but the effectiveness of the intervention supports secondary prevention as an important component of existing healthcare cardiac rehabilitation measures (40). Subsequently, it can be applied to the full spectrum of coronary syndromes and to the rehabilitation of patients with other related conditions.

## 5 Conclusion

The study found that the use of secondary prevention by various rct studies in postoperative patients with PCI was not limited to medications, but the five nonpharmacological measures of patient education, exercise, dietary modification, blood pressure control, and smoking cessation were also actively used. Some studies have combined mHealth technology with secondary prevention measures and found that the synergistic effect of mHealth on the interventions is considerable, especially the interactive feature of mHealth deserves to be focused on in later studies.

However, we found that the changes in objective prognostic indicators of postoperative PCI patients using secondary prevention interventions were not significant, and the outcomes were not significant. Although subjective indicators such as lifestyle were further improved, objective measures are truly important indicators of whether the disease has been improved, which is an issue that deserves to be emphasized in subsequent studies. Meanwhile, educational, economic, and social support challenges within the geriatric population may impede the effective delivery of secondary prevention consequently, future research should prioritize addressing these issues to optimize outcomes for older adult patients.
