# Supplementary material for: Using secondary prevention strategies in patients after PCI: a narrative review
Source: Front Public Health. 2025 Jul 10;13:1562201. doi: 10.3389/fpubh.2025.1562201 (PMC12286798; doi:10.3389/fpubh.2025.1562201)
Supplement: Supplementary file 1 [file Supplementary_file_1.docx]

**Supplementary Table 1** Results of randomised controlled trials (Rcts) quality assessments

| **Included literature** | **Items** | | | | | | | | | | | | | **Inclusion or not** |
| --- | --- | --- | --- | --- | --- | --- | --- | --- | --- | --- | --- | --- | --- | --- |
|  | **1** | **2** | **3** | **4** | **5** | **6** | **7** | **8** | **9** | **10** | **11** | **12** | **13** |  |
| **B. M. A. van Bakel et al.** ^[34]^ | Y | Y | Y | N | N | Y | Y | Y | Y | Y | Y | Y | Y | Y |
| **Natássia Condilo Pitta et al.** ^[37]^ | Y | Y | Y | N | N | Y | Y | Y | Y | Y | Y | Y | Y | Y |
| **Jang-Whan Bae et al.** ^[24]^ | Y | Y | Y | N | N | Y | Y | Y | Y | Y | Y | Y | Y | Y |
| **Christel Bruggmann et al.** . ^[21]^ | Y | Y | Y | Not clear | N | Y | Y | Y | Y | Y | Y | Y | Y | Y |
| **Oscar Hägglund et al.** ^[39]^ | Y | Y | Y | N | N | Not clear | N | Y | Y | Y | Y | Y | Y | Y |
| **Alyna Turner et al.** ^[35]^ | Not clear | Y | Y | Not clear | Not clear | Y | Y | Y | Y | Y | Y | Y | Y | Y |
| **Stéphane Rinfret et al.** ^[36]^ | Y | Y | Y | N | N | Y | Y | Y | Y | Y | Y | Y | Y | Y |
| **Barbara M. Murphy et al.** | Not clear | Not clear | Not clear | Not clear | Not clear | Not clear | Not clear | Not clear | Not clear | Y | Y | Y | N | N |
| **Holli A. DeVon et al.** ^[40]^ | Y | Y | Y | Not clear | Not clear | Y | Y | Y | Y | Y | Y | Y | Y | Y |
| **Jan Lisspers et al.** ^[38]^ | Y | Not clear | Y | Not clear | Not clear | Not clear | Y | Y | Y | Y | Y | Y | Y | Y |

Y：Yes；N：No

Notice：1 Whether the allocation of participants was actually randomized；2 Whether the grouping scenario is taken for assignment hiding；3 Whether the baseline of the experimental group and the control group is comparable；4 Whether the study participants were blinded；5 Whether the interventionist was blinded；6 Whether the outcome evaluators were blinded；7 In addition to the intervention to be verified, whether the other measures received by the groups were the same；8 Whether the follow-up is complete and, if not, whether measures are taken to deal with it；Other: 9 Whether all enrolled study subjects were included in the outcome analysis；10 Outcomes were assessed in the same way across groups；11 Reliability of the methods used to measure outcomes；12 Appropriateness of the data analysis methodology；13 Whether the study design was sound and whether there were deviations from the standard RCTs in the conduct and analysis of the study


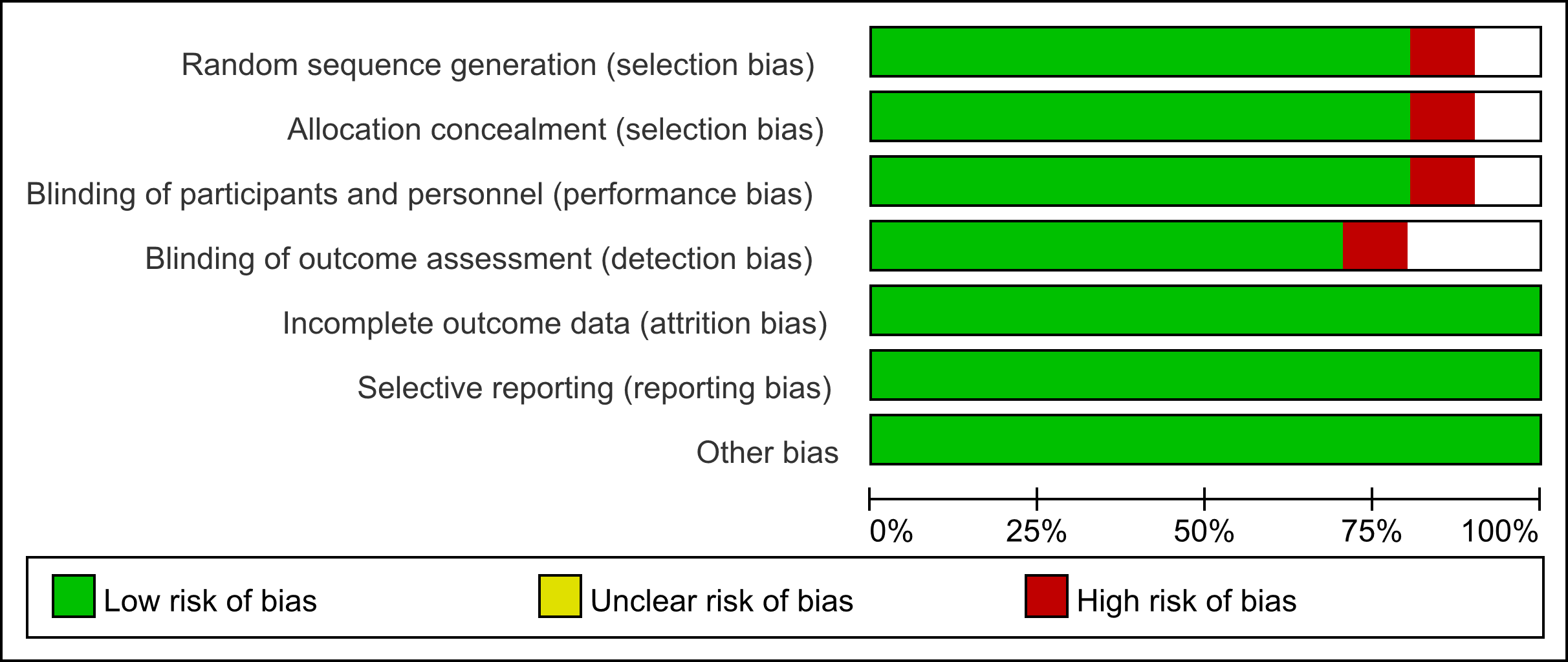


**Supplementary Figure 1** Literature Quality Evaluation Form


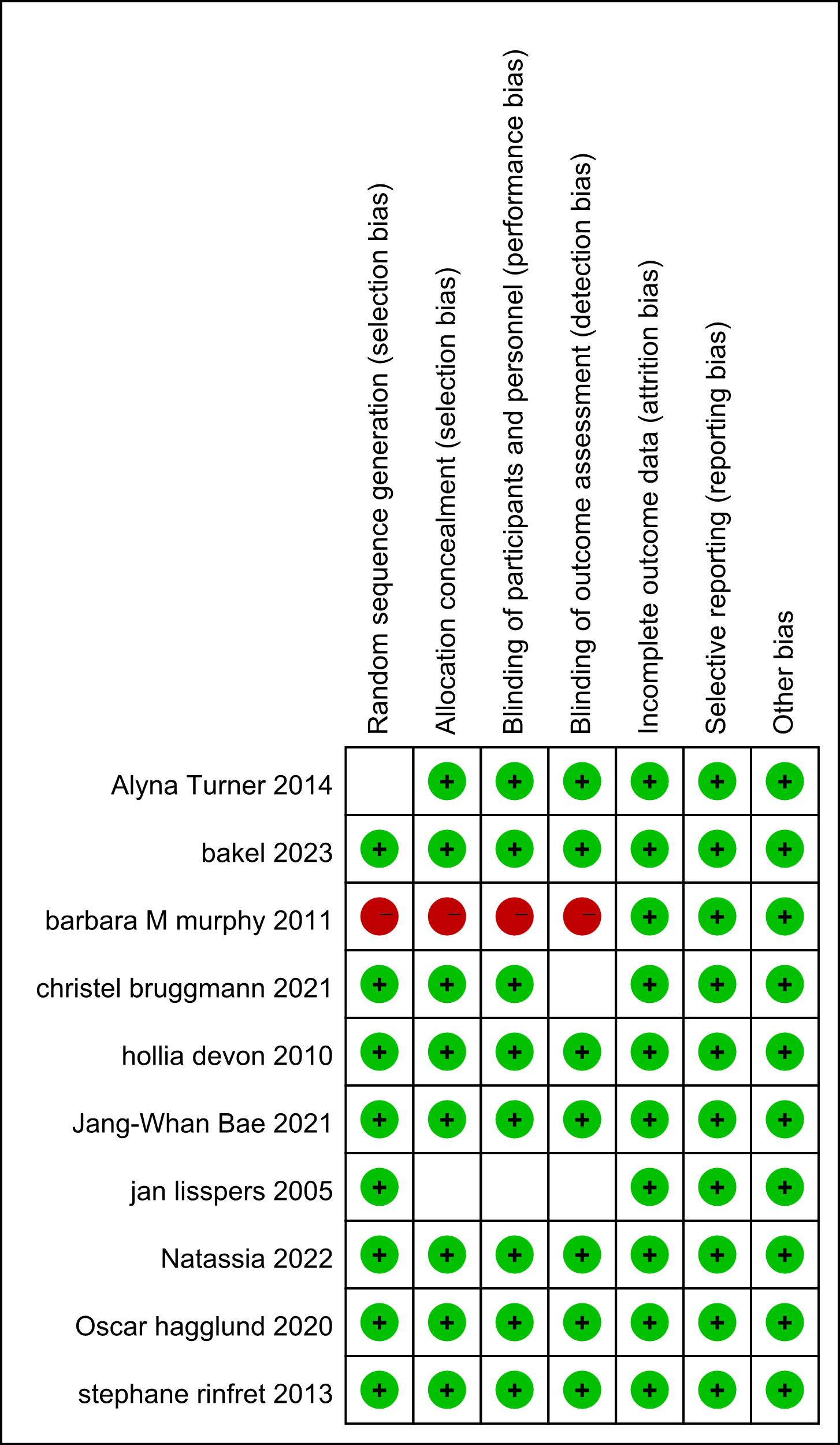


**Supplementary Figure 2** Literature Quality Evaluation Form
